# Attitude of Albanian psychiatrists towards their patients

**DOI:** 10.1192/j.eurpsy.2023.556

**Published:** 2023-07-19

**Authors:** E. Dashi, V. Skendi, D. Ori, V. Alikaj

**Affiliations:** ^1^Department of Neuroscience, University Hospital “Mother Tereza”, Tirane, Albania; ^2^ Institute of Behavioural Sciences, Semmelweis University; ^3^Department of Mental Health, Heim Pal National Pediatric Institute, Budapest, Hungary; ^4^Faculty of Medicine, Tirana Medical University, Tirane, Albania

## Abstract

**Introduction:**

Erving Goffman described stigma as an attribute considered to be undesirable and unpleasant by society and which differentiates the stigmatised person from other members of the community that he or she should belong to. (Hankir AK, et al., 2014). Mental illness has been always associated with stigma, moreover people with serious mental illness have higher rates of mortality and morbidity. (Patrick W. Corrigan, et al., 2014). The contact mental-health-care professionals have with people with mental illness is associated with positive attitudes towards civil rights, however it does not reduce stigma as does social contact such as with friends or family members with mental illness. (Henderson C et al., 2014)

**Objectives:**

This is the Albanian substudy of a larger multicenter study. We aimed to investigate the attitudes of specialists and trainees in psychiatry in Albania.

**Methods:**

An anonymous online questionnaire was sent by email to the participants. We used questions on sociodemographic and professional details as well as requested personal information regarding their lived experience. The self reporting Opening Minds Stigma Scale for Health Care Providers was used to measure stigmatising attitudes, which contains 15-statements and 3-subscales: Attitude, Disclosure and Help-seeking and Social distance.

**Results:**

Altogether 59 professionals completed the questionnaire, 59% of them worked as adult (n=35) and 41% as child psychiatrists (n=24). 58% were specialists (n=34) and 41% trainees (n=24). Based on their responses, 12% of them (n=7) have ever sought help for their own mental health problems. Regarding case discussion, supervision or Balint groups, 81% of the sample (n=48) was open to these; however, it was accessible for only 46% of the sample.

The median stigma scores were the followings: attitude: 13 (11-16), Disclosure: 10 (9-12) and Social distance: 12 (9-13), total score: 35 (31-40); however, none of these were associated with any of the above variables.

**Conclusions:**

Stigma is present towards people with mental health problems and psychiatrist play their part in it as well. Further investigation is needed into Albanian psychiatrists’ stigmatising attitudes to find appropriate anti-stigma interventions for them.

**Disclosure of Interest:**

None Declared

